# Impact of breast cancer genetic testing in Brazilian patients: insights from the MAGENTA study

**DOI:** 10.3389/fonc.2025.1597891

**Published:** 2026-01-21

**Authors:** Evelin Scarelli, Carolina Meyn Teixeira, Nathália Viana e Silva, Andrea Silveira dos Santos Bredariol, Giovana Sivieri Baracho, Fabiano Hahn Souza, Luciana Holtz

**Affiliations:** 1 Instituto Oncoguia, São Paulo, Brazil; 2 AstraZeneca Brasil, São Paulo, Brazil; 3 Corebox, São Paulo, Brazil

**Keywords:** breast cancer, genetic testing, oncogenetics, patient perspective, precision oncology, BRCA testing

## Abstract

**Introduction:**

Breast cancer is the most prevalent cancer among women in Brazil, with up to 10% of cases linked to hereditary factors. Genetic testing and counseling are critical for identifying hereditary breast cancer risk, guiding treatment decisions, and preventing cancer in high-risk populations.

**Objectives:**

This study aimed to analyze the experience, perspectives, and access barriers to breast cancer genetic testing in the Brazilian respondents of the Multinational Survey Study Assessing GENetic Testing and Counseling Among Patients with Breast Cancer (MAGENTA) study.

**Methods:**

A 38-multiple-choice question, branched survey was distributed by patient advocacy agencies to collect sociodemographic and patient-perspective data about their experience with breast cancer genetic testing. A multivariate analysis was conducted to explore the association between sociodemographic variables and the genetic testing status.

**Results:**

207 breast cancer patients completed the survey. In this cohort, the rates of genetic testing and counseling were 81.6% and 60.4%, respectively. 66.7% of respondents reported a high or advanced educational level. Notably, 71.7% of patients reported that genetic testing changed their treatment plans, and 77.1% of those who took the test were willing to test their familiars. In addition, 98.7% stated they did not regret undergoing genetic testing. Higher income was independently associated with higher odds of undergoing genetic testing (OR: 4.43 [95% CI: 1.64;13.11]; p = 0.0011), while having more than 50 years-old was associated with a lower odds of undergoing testing (OR: 0.21 [95% CI: 0.08;0.56]; p=0.0018). Barriers such as costs and limited awareness were prominent, with 89% of patients in Brazil reporting low awareness of genetic testing prior to their diagnosis.

**Conclusions:**

The survey respondents in Brazil comprised a highly educated and financially secure group of patients. Although not generalizable to the entire Brazilian population, our results revealed that even in a highly educated and well-informed cohort there is a strong association between age and income level with genetic testing. These findings expose the real-world challenges for increasing genetic testing coverage in Brazil, where testing is only warranted in the private health system, highlighting the need for health policies to increase test availability for lower income brackets.

## 1 Introduction

Breast cancer (BC) is the most frequent cancer among women globally (1). In Brazil, approximately 73,000 women receive a breast cancer diagnosis each year (2). Genetically inherited cancers represent up to 10% of all cases and are often associated with pathogenic or likely pathogenic (P/LP) variants in predisposition genes (3). The *BRCA1* and *BRCA2* genes harbor the most common hereditary variants, although the genetic landscape in Brazil presents distinct features, such as a higher prevalence of *TP53* mutations (4, 5). Notably, over 50% of early-onset BC cases with *BRCA1/2* mutations report no family history, underscoring the importance of genetic testing beyond traditional risk assessment based on ancestry (6).

International guidelines recommend genetic testing and counseling for high-risk individuals to improve clinical decisions and manage cancer risk (7). For instance, the American Society of Clinical Oncology (ASCO) recommends *BRCA1/2* mutation testing for all newly-diagnosed BC patients aged 65 or younger, as well as for patients over 65 who have additional risk factors (8). Testing is also critical for women of any age with specific histological features, such as triple-negative BC, which do not express hormone receptors or HER2, to guide specific treatments (9). To understand the implementation of these guidelines in Brazil, it is crucial to recognize its dual healthcare structure. The public system, known as Unified Health System (SUS), is a government-funded universal healthcare system that covers approximately 75% of the population. The remaining 25% are served by a supplementary private healthcare sector funded by health insurance plans (10).

This division creates significant disparities in access to medical services, including genetic testing. In the private sector, genetic testing for *BRCA1/2* is included in the list of mandatory procedures covered by insurance plans for any diagnosed breast cancer in male or any women under 35 years old, at least two breast cancers under 50 years, triple negative cancers under 60 years, or strong family history. However, this coverage is often hindered by bureaucratic barriers, such as the requirement for the test to be requested by a medical geneticist (11). Within the SUS, there is no reimbursement codification for breast cancer genetic testing, and it is not routinely offered, being restricted to tertiary and quaternary centers linked to research projects. Despite the international recommendations, for most of the Brazilian population reliant on the public system, access to genetic testing and counseling remains unavailable, creating a significant equity gap in precision oncology and preventative care (12, 13).

As a positive *BRCA1/2* test result serves as a critical biomarker to patients whose tumors are susceptible to targeted therapies, the consequence of this testing gap is the systematic exclusion of patients, especially those from SUS, from the benefits of precision oncology. Ideally, patients should be informed about the benefits, limitations, and different types of results, including positive, negative, and variants of uncertain significance (VUS) (14). A qualified oncogenetics professional should provide pre- and post-testing counseling, but the availability of these services are hindered by socioeconomic factors, low awareness, and insufficient healthcare resources (8, 9, 15).

In order to understand the barriers that limit access to breast cancer genetic services in Brazil, it is crucial to understand the patients’ perspective of the benefits of the test. These perspectives should be taken in consideration when developing strategies to raise awareness among the general population about hereditary cancer, the importance of family history, and the availability of genetic counseling and testing. In this regard, the Multinational Survey Study Assessing GENetic Testing and Counseling Among Patients with Breast Cancer (MAGENTA) was conducted to gain a comprehensive understanding of patient experiences and to identify challenges in accessing these services (16).

The Brazilian cohort of the MAGENTA survey was conceived within this context of disparity. This study analyzed the experience and perspectives of breast cancer genetic testing in a group of patients with comparatively high access to information and services, primarily composed of patients highly engaged with patient advocacy networks. By focusing on this demographic, we aimed to characterize an ‘ideal scenario’ for genetic testing.

## 2 Methods

### 2.1 Survey design

This study was based on the Multinational survey study Assessing GENetic Testing MAGENTA (16). The survey was developed by the Genetic Testing and Breast Cancer-Patient Author Steering Committee, which comprised patient authors with a personal history of breast cancer from nine countries: Argentina, Australia, Brazil, Egypt, India, Malaysia, Mexico, Russia, and Taiwan. The committee convened to identify key themes and regional barriers related to the genetic testing and counselling landscape.

**Figure 1:**
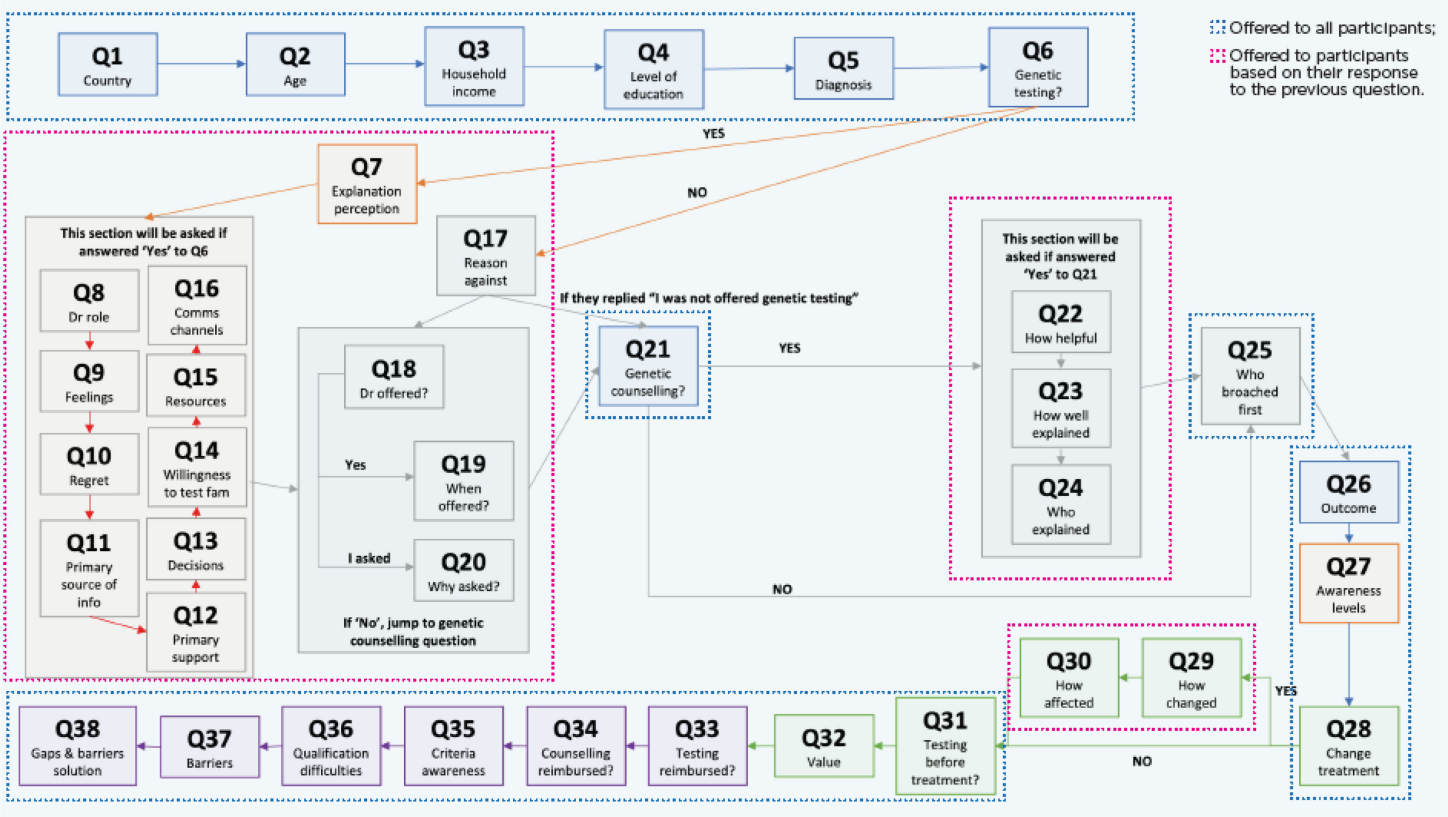
Patient survey flowchart. The flowchart shown here demonstrates the sequence of questions encountered by a survey participant. Level 1 questions (boxed in blue) appeared in the survey flow of all participants. Level 2 questions (boxed in pink) appeared in the survey flow, depending on the response to the previous question.

A 38-item, multiple-choice questionnaire was drafted in English and subsequently translated into Portuguese for dissemination. The full list of survey questions is provided in **Table 1**. Response options were primarily fixed choices, with some questions allowing for single or multiple selections as specified. Briefly, questions 1–5 were designed to collect sociodemographic data, and question 6 asked about the genetic testing status (yes/no). The remaining questions (7-38) were designed to assess the patients’ experience and perspectives about breast cancer genetic testing. A key feature of the survey design was the use of survey branching, which created an individualized question pathway for participants. As illustrated in the flowchart in **Figure 1**, the questions a participant received depended on their answers to preceding questions. Consequently, the number of respondents varied across different questions of the survey. The MAGENTA survey instrument, applied globally, was not formally validated for the Brazilian population. This approach was intentionally maintained to ensure data standardization across the different countries where the survey was conducted.

**Table 1:** List of questions provided to survey participants.

N°	Question	Response options
1	Which country are you from?	• Brazil
2	How old are you? Please specify in years.	0-120
3	What is your household income?	• Up to 3SM (R$3636) (RF1) • 3–5 SM (R$ 3636-6060) (RF2) • 5–10 SM (R$ 6060-12.120) (RF3) • 10–20 SM(R$12.120-24.240) (RF4) • >20 SM (>R$ 24.240) (RF5) • I prefer not to disclose (RF0)
4	What is your highest level of education?	• Some high school (E1) • High school diploma (E2) • Some college (E2) • Trade/technical/vocational training (E3) • Associate’s degree (E3) • Bachelor’s degree (E3) • Master’s degree (E4) • Professional degree (E4) • Doctorate (E4)
5	How old were you when you were diagnosed with breast cancer?	–
6	Have you undergone genetic testing?	• Yes • No
7	What is your perception of how knowledgeable your doctor is in explaining genetic testing and its implications to you?	• Very knowledgeable • Knowledgeable • Somewhat knowledgeable • Less knowledgeable • Not knowledgeable
8	What was the role of your oncologist through the genetic testing process?	• My oncologist referred me for genetic testing, explained the process and results to me throughout the process, and used the result to inform treatment selection • My oncologist referred me for genetic testing, explained the process to me at the beginning, and used the result to inform treatment selection • My oncologist referred me for genetic testing and used the result to inform treatment selection • Not applicable • Other (please specify)
9	How did you feel through the genetic testing process? Please select up to 3 only.	Afraid Angry Anxious Ambivalent Apprehensive Concerned Depressed Grateful Guilty Hopeful Isolated Overwhelmed Surprised Other (please specify)
10	Do you regret undergoing genetic testing?	• Yes • No
11	Who was your primary source of information during the genetic testing process?	Oncologist Nurse Genetic counsellor Patient support groups Family members Other (please specify)
12	Who was your primary source of psychological support during the genetic testing process?	Oncologist Nurse Genetic counsellor Patient support groups Family members Other (please specify)
13	How did you come to decide to undergo genetic testing?	• After speaking with my doctor/genetic counsellor • After reading up on the internet • After consulting with my family • After another family member was diagnosed • After consulting with patient support groups, including non-governmental organizations, Facebook/social media groups, online forums, etc. • Other (please specify)
14	How willing would you be to have your children and other family members undergo genetic testing?	• Very willing • Willing • Somewhat willing • Less willing • Not willing
15	What resources, beyond your oncologist and/or doctor, were available to you to guide your genetic testing experience? Please select up to 3 only.	• Patient support groups • Genetic counselling • Brochures/pamphlets • Websites • Facebook/social media groups • Other (please specify)
16	How do you prefer to receive information? Please select up to 3 only.	• Printed materials • Websites • Email • Messaging apps, e.g., WhatsApp, Viber, Line, etc. • Face-to-face • Phone calls • Video chats • Podcasts • Chatbots • Other (please specify)
17	What was the main reason for deciding against undergoing genetic testing?	• I was not offered genetic testing • I was afraid of what the genetic test results would be • I did not qualify for genetic testing reimbursement • Cost of genetic testing • Life insurance implications • Social stigma from family/community/potential employers • Other (please specify)
18	Were you offered genetic testing by your doctor?	• Yes • No • I asked for genetic testing
19	When were you offered genetic testing by your doctor?	Before diagnosis At diagnosis During treatment After treatment failure After relapse I had to ask for genetic testing
20	Why did you ask for genetic testing?	• I have known family medical history • I have known risk factors • I qualify for genetic testing • I just wanted to know • Other (please specify)
21	Did you receive any genetic counselling?	• Yes • No • I am not sure
22	How helpful was genetic counselling in clarifying the genetic testing process and its implications?	• Very helpful • Helpful • Somewhat helpful • Less helpful • Not helpful
23	How well was the genetic testing process and result implications explained to you by your doctor or genetic counsellor?	• I fully understood the process and implications • I understood most of the process and implications • I understood some of the process and implications • I understood little of the process and implications
24	Who provided genetic counselling or explained the implications of your genetic testing results to your family?	• My medical oncologist • My surgeon • My GP or other doctor • A genetic counsellor • A clinic nurse • A psychologist • Myself • Nobody • Other (please specify)
25	Who first raised the discussion of genetic testing and counselling with you?	My medical oncologist My surgeon My GP or other doctor A genetic counsellor A clinic nurse Patient support group Friend or family member It was never broached with me Other (please specify)
26	What was the outcome of your genetic test?	*BRCA1*-positive *BRCA2*-positive *BRCA*-negative I have not undergone genetic testing Other (please specify)
27	How would you rate the awareness levels of genetic testing and counselling of the following persons and populations? Yourself, before diagnosis Your doctor Your community	Very high High Moderate Low Very low
28	Did genetic testing change the treatment strategy for your breast cancer?	Yes No
29	How did genetic testing change your treatment strategy? Select all that apply.	Unilateral mastectomy to bilateral mastectomy Chemotherapy/radiotherapy to targeted therapy Addition of treatment pre- or post-surgery Addition of surgery pre- or post-treatment Requiring a second surgery Other (please specify)
30	How did the changes in treatment strategy affect you? Select all that apply.	Treatment delays due to complexities in switching Psychological stress Cost implications No effect Other (please specify)
31	In your opinion, should all patients diagnosed with breast cancer undergo genetic testing first before starting treatment?	Yes No, only patients with a family history of breast cancer or other risk factors should undergo genetic testing No
32	What value(s) do you see/have you experienced with genetic testing? Please select up to two only.	To inform treatment decisions Surveillance and early detection of breast cancer in family members Awareness for family planning There is no value Other (please specify)
33	Should you qualify, is the cost of genetic testing for breast cancer reimbursed in your country?	Yes, fully Yes, partially No I don’t know
34	Should you qualify, is the cost of genetic counselling for breast cancer reimbursed in your country?	Yes, fully Yes, partially No I don’t know
35	Do you have knowledge and understanding of the criteria to qualify for genetic testing in your country?	Yes No I don’t know
36	In your opinion, how difficult is it to qualify for genetic testing to be reimbursed in your country?	Very difficult Difficult Neither difficult nor easy Easy Very easy I don’t know Please specify the reason for your answer.
37	In your opinion, what are the main barriers to genetic testing for you and your family? Please select up to 2 only.	Cost Lack of understanding Social stigma and discrimination Family objections Other (please specify)
38	In your opinion, what is a solution that would best address the gaps and overcome the barriers to genetic testing and counselling in your country?	Public awareness campaigns to raise patient and community awareness of the value of genetic testing and counselling for breast cancer Healthcare professional education to increase knowledge and understanding of genetic testing and counselling and its implications in breast cancer treatment Updating clinical guidelines to include genetic testing and counselling as part of the treatment and management of breast cancer patients Facilitating the qualification of genetic testing and counselling reimbursement for breast cancer patients Legislation to protect patients who are genetic mutation carriers from discrimination Additional educational/informational resources to empower patients and their families on genetic testing and counselling • Other (please specify)

The survey was conducted through the Within3 (https://within3.com/) platform and made available to the survey participants. The default number of responses for questions accompanied by response options is 1, with some questions allowing the selection of multiple responses, as specified in the question. SM, minimum wage; GP, general practitioner; *BRCA*, breast cancer gene.

### 2.2 Sampling

The MAGENTA survey was an open, free intention questionnaire distributed by local patient advocacy groups, which localized and disseminated the survey through their social media and patient platforms. In Brazil, the survey was distributed by the Oncoguia Institute (17) and the Brazilian Federation of Philanthropic Institutions Supporting Breast Health (FEMAMA). Therefore, we did not estimate a required sample size before the distribution of the survey. The dissemination of the survey reached a total of 207 patients who answered the questionnaire, thus constituting the cohort of this study.

### 2.3 Statistical analysis

The categorical data for each question were reported as absolute (n) and relative (%) frequencies. All statistical analyses were performed using the stats package in the R software (version 4.5.1) (18, 19). Fisher’s exact tests were used to assess the bivariate association between sociodemographic characteristics (age group, income level, and education) and the genetic testing status (yes/no) using the fisher.test() function. To determine factors independently associated with genetic testing in this cohort, we conducted an exploratory multivariate analysis using a logistic regression model via the glm() function with the ‘family’ parameter set to family = binomial(link = “logit”). The dependent variable was genetic testing status (yes/no), and the independent variables included in the model were those that demonstrated a significant association (p < 0.05) in the bivariate analysis. The results of the regression model are presented as Odds Ratios (OR) with their respective 95% Confidence Intervals (95% CI). Associations presenting a p < 0.05 were considered significant.

## 3 Results

### 3.1 Participant characteristics

A total of 207 individuals from Brazil participated in the survey, accounting for 13.6% of the 1,524 total respondents in the global MAGENTA survey. The number of respondents varies for specific questions throughout this section due to survey branching, where participants were directed to different question sets based on their previous answers. The survey responses for all participants are presented in **Table 2**.

**Table 2:** Demographic characteristics of survey respondents, according to their geographic localization.

Characteristic	Brazil	Global^║^
Brazil regions, n (%)*		
Southeast	127 (62%)	–
South	31 (15%)	–
North East	22 (11%)	–
Midwest	14 (7%)	–
North	10 (5%)	–
Age group at time of survey, n(%)		
Median age [years (IQR)]	40 (35-46)	47 (39-55)
18–44 years	145 (70%)	383 (39.5%)
45–64 years	61 (29.5%)	495 (51.1%)
65–74 years	1 (0.5%)	86 (8.9%)
>75 years	0	5 (0.5%)
Income group, n (%)^Δ^		
Low	67 (32.3%)	226 (19.2%)
Medium	72 (34.8%)	281 (23.9%)
High	35 (16.9%)	32 (2.7%)
Prefer not to say	33 (16%)	430 (36.5%)
Education level, n (%)^Δ^		
Low	7 (3.4%)	134 (13.8%)
Medium	62 (29.9%)	161 (16.6%)
High	91 (44%)	537 (55.5%)
Advanced	47 (22.7%)	132 (13.6%)
Prefer not to say	0	5 (0.5%)
Underwent genetic testing		
Yes	169 (81.6%)	568 (58.6%)
No	38 (18.4%)	401 (41.4%)
Underwent genetic counseling		
Yes	113 (60.4%)	245 (29%)
No	55 (29.4%)	488 (57.7%)
Not sure	19 (10.2%)	113 (13.3%)
Levels of awareness of genetic testing and genetic counseling of survey participants (pre-diagnosis)		
Very high or high	20 (11%)	283 (32.1%)
Moderate to very low	161 (89%)	597 (67.9%)
Levels of awareness of genetic testing and genetic counseling of physicians (patient perspective)		
Very high or high	139 (76.8%)	300 (44.3%)
Moderate to very low	42 (23.2%)	377 (55.7%)
Levels of awareness of genetic testing and genetic counseling of the community (patient perspective)		
Very high or high	18 (10%)	58 (8.6%)
Moderate to very low	163 (90%)	617 (93.4%)

*Numbers are rounded; ^║^Data adapted from Powel et al. (16); **^Δ^**Women in Brazil were left out of count for global column. Countries represented in the global column: Argentina, Australia, Egypt, India, Malaysia, Mexico, Russia and Taiwan.

The Brazilian cohort was, on average, younger than the global cohort, with a median age of 40 compared to 47 years, respectively. A large proportion of the Brazilian participants (70%) were in the 18–44 age group, whereas this group represented 39.5% of the global participants. Respondents from Brazil also reported higher levels of education compared to their global counterparts. The majority of Brazilian respondents were from the Southeast region (62%).

### 3.2 Genetic testing and counseling uptake in the Brazilian respondents

In Brazil, 81% (n=169) of respondents had undergone genetic testing, and 60.4% (n=113) had received genetic counseling. These rates were higher in Brazil than in the global cohort, where 58.6% reported undergoing testing and 57.7% received counseling. Regarding the age group of the patients within the Brazilian cohort, 88.5% of women under 45 had been tested, compared to only 11.5% of those aged 45 and older. In the bivariate analysis model, age group, income level, and education level were significantly associated with undergoing genetic testing (**Table 3**). In the multivariate analysis corrected by age group, income, and education level, the odds of undergoing genetic testing in patients with more than 50 years-old was 79% lower compared to patients with less than 50 years-old (OR: 0.21 [95% CI: 0.08;0.56]; p=0.0018). Conversely, respondents with a household income above 3 minimum-wages had an odds of undergoing genetic testing more than 4-times higher than those with a household income below 3 minimum wages (OR: 4.43 [95% CI: 1.64;13.11]; p = 0.0011) (**Table 4**). Other sociodemographic factors did not show a significant association with the odds of undergoing genetic testing in this cohort.

**Table 3:** Bivariate analysis of the association between sociodemographic characteristics and genetic testing status.

Variable	Category	Genetic testing status N (%)		p-value
		Positive	Negative	
Age group	≤50 years-old	154 (74.4)	28 (13.52)	0.0017
	>50 years-old	14 (6.77)	11 (5.31)	
Education level	E1	3 (1.44)	4 (1.93)	0.0021
	E2	44 (21.26)	18 (8.69)	
	E3	78 (37.7)	13 (6.28)	
	E4	43 (20.77)	4 (1.93)	
Household income level	RF0	21 (10.14)	12 (5.79)	<0.0001
	RF1	47 (22.7)	20 (9.66)	
	RF2+	100 (48.3)	7 (3.38)	

E1, Some high school; E2, High school diploma or Some college; E3, Trade/technical/vocational training or Associate’s degree or Bachelor’s degree; E4, Master’s degree or Professional degree or Doctorate. RF0, prefer not to disclose; RF1, up to 3 minimum wages; RF2+, more than 3 minimum wages.

**Table 4:** Multivariate analysis of the association between sociodemographic characteristics and genetic testing status.

Variable	Category	Genetic testing status N (%)		OR	95% CI	P-value
		Positive	Negative			
Age group	≤50 years-old	154 (74.4)	28 (13.52)	Ref	–	–
	>50 years-old	14 (6.77)	11 (5.31)	0.21	0.08; 0.56	0.0018
Education level	E1	3 (1.44)	4 (1.93)	Ref	–	–
	E2	44 (21.26)	18 (8.69)	2.53	0.48; 14.57	0.2710
	E3	78 (37.7)	13 (6.28)	4.48	0.81; 27.19	0.0840
	E4	43 (20.77)	4 (1.93)	5.59	0.81; 44.16	0.0844
Household Income	RF1	47 (22.7)	20 (9.66)	Ref	–	–
	RF2+	100 (48.3)	7 (3.38)	4.43	1.64; 13.11	0.0045

E1, Some high school; E2, High school diploma or Some college; E3, Trade/technical/vocational training or Associate’s degree or Bachelor’s degree; E4, Master’s degree or Professional degree or Doctorate. RF0, prefer not to disclose; RF1, up to 3 minimum wages; RF2+, more than 3 minimum wages.

Most genetic tests (46.3%) were performed at the time of the breast cancer diagnosis. Awareness levels prior to diagnosis were generally low. Among 181 Brazilian respondents, 89% reported their own awareness as “moderate to very low,” and 90% perceived community-level awareness to be in the same range. In contrast, participants perceived their physicians’ awareness as high, with 76.8% rating it as “high or very high” (**Table 2**).

### 3.3 Role of the oncologist and impact on treatment

Most patients (69% of 153) stated that their oncologist referred them for genetic testing and subsequently used the results to guide treatment decisions. This was more frequent than in other countries, where only 21.7% of patients reported the same experience. However, the survey also revealed that more than half of the patients were not offered the option of undergoing genetic testing.

Genetic test results led to a change in the treatment strategy for 71.7% of Brazilian patients, compared to 44.7% of global respondents (**Table 5**). The most common treatment modification in Brazil was changing the surgical plan from a unilateral to a bilateral mastectomy, which occurred in 67.2% of cases (82 out of 122 patients). This was also the most frequent alteration globally, but at a lower rate of 37.5%. Other treatment changes included a switch to targeted therapy (23.8%) and the addition of a second surgery (27%).

**Table 5:** Impact of genetic testing on breast cancer patient treatment.

Treatment change	Brazil (n=209)	Global^Δ^ (n) (n=530)
Unilateral to bilateral mastectomy	67.2% (82)	36.7% (194)
CT/RT to target therapy	23.8% (29)	25.8% (136)
Addition of treatment pre- or post-surgery	13.9% (17)	36.4% (192)
Addition of surgery pre- or post-treatment	15.6% (19)	10.5% (55)
Requiring second surgery	27% (33)	4.2% (22)

**^Δ^**Women in Brazil were left out of count for global column. Countries represented in the global column, Argentina, Australia, Egypt, India, Malaysia, Mexico, Russia and Taiwan; CT, chemotherapy; and RT, radiotherapy.

### 3.4 Perception of benefits and barriers to accessing genetic testing

Respondents in Brazil were “very willing” to have their family members and children tested (77.1%), a significantly higher rate than the global population (43.7%). A majority of the Brazilian sample (66.3%) agreed that testing was beneficial for the surveillance and early detection of breast cancer in their relatives. Most respondents (98.7%) reported having no regrets about their decision to undergo testing, similar to global respondents (98.5%). Genetic counseling was considered helpful by 89.3% of those who received it. This counseling was most often provided by a genetic counselor (34.8%) or an oncologist (30.4%).

The primary perceived barriers to accessing genetic testing were cost and a lack of understanding of the implications of the tests. Cost was cited as a key barrier by 81.3% of Brazilian respondents. This was compounded by a lack of clarity on financial coverage; approximately 60% of women who qualified for testing reported they were either not informed about reimbursement or lacked this information. Only 36.7% expressed confidence in their understanding of reimbursement criteria, and 30.1% were uncertain about their coverage status. To improve access, 79.5% of respondents recommended that all breast cancer patients should undergo genetic testing before beginning therapy. Other suggested approaches included changing clinical guidelines, initiating public awareness campaigns, and enhancing the education of healthcare practitioners.

## 4 Discussion

This study provides initial insights into the genetic testing experience in Brazil by focusing on a subgroup of patients with high levels of information, engagement, and access to healthcare resources. The dissemination of the survey through patient advocacy groups predominantly reached a highly engaged and digitally literate audience, capturing a profile of informed and proactive patients, allowing to study the perceptions and impacts of genetic testing when barriers of information and engagement are overcome. This socioeconomic profile, while not representative of the general population, represents a group with favorable conditions such as access to information and resources for active engagement in their health journey. Therefore, the results we presented here should not be generalized to the broader Brazilian population. Despite the limitations of the survey, which are discussed in detail later, our findings serve as a case study on the potential benefits of genetic testing when access barriers to healthcare resources and quality information are minimized.

The high rates of testing uptake (81.6%) observed in this cohort, substantially higher than the global MAGENTA study average (58.6%), cannot be dissociated from the respondents’ proactive profile (16). Although pre-diagnosis awareness was notably low (89%), the post-diagnosis pursuit of support groups and information indicates a patient profile that actively seeks support and information. This proactivity is crucial, as it directs the patient towards interaction with qualified healthcare professionals. The decision to test was made by 66.7% of patients after dialogue with their physician or genetic counselor, underscoring the critical importance of professional guidance, whether from an oncologist, mastologist (breast surgeon), or geneticist, to transform patient proactivity into decisive clinical action.

Given the shortage of geneticists in Brazil, other physicians, such as oncologists, surgeons, and mastologists (breast surgeons) can play a key role in requesting testing and counseling patients and their families (8, 9). Brazilian respondents were more likely to report that their oncologist was instrumental in referring them for genetic testing and in using the results to guide treatment decisions. The results reinforce that genetic testing led to changes in treatment strategies for many respondents, with the most common alteration being a switch from unilateral to bilateral mastectomy (67.2%) (20). However, the survey did not investigate whether these surgical decisions were specifically linked to patients who tested positive for a pathogenic mutation.

Respondents were also more inclined to have their family members tested, indicating a willingness to proactively mitigate health risks among their families. Proactive testing facilitates early detection and allows individuals and families to make informed health decisions (21, 22). The majority of respondents found genetic counseling and testing valuable, despite the negative emotions experienced during the process. These positive perceptions reinforce the need for personalized risk assessments and action plans (23, 24).

A significant barrier identified by this survey was the low awareness of genetic testing before a breast cancer diagnosis, which limits its potential benefits in primary and secondary cancer prevention. Importantly, more than half of the patients were not offered the option of undergoing genetic testing. This underscores the need for extensive training and education for doctors on the benefits of genetic testing and counseling. Given the limited availability of genetic counselors in Brazil, oncologists and surgeons must be equipped with adequate training to provide appropriate counseling to their patients (15).

Regarding costs, approximately 60% of women reported they were either not informed about reimbursement for the genetic test in the private sector or lacked this information. Despite these challenges, around 99% of Brazilian respondents reported having no regrets about undergoing genetic testing, highlighting its perceived benefits. The identification of BC risk mutations facilitates the monitoring of high-risk family members, enables personalized treatments, and supports more assertive surgical interventions.

Our results were observed in a group of patients defined by a higher income bracket, likely with more access to healthcare resources and patient advocacy support. Although the survey did not directly inquire whether patients received treatment within the public system (SUS) or the supplementary private sector, the characteristics of the MAGENTA cohort in Brazil match with the private criteria for hereditary breast cancer testing, such as a diagnosis under the age of 35, occurrence of two breast cancers under 50, and triple-negative breast cancer under 60 years-old. This patient profile, which also presented a high prevalence of household income above 3 minimum wages, suggests that access to breast cancer testing in Brazil remains restricted to the minority of the population with access to the private healthcare system. In fact, respondents with a household income above 3 minimum wages had over 4-times the odds of undergoing testing (**Table 4**), reinforcing that the bias observed in our sample is a reflection of the socioeconomic reality in Brazil and the lack of availability of genetic testing in the public system. These results highlight that public policies aiming to replicate the positive outcomes of this case study must first consider financial disparities and the availability of genetic testing in the public system as the main barrier to equity in precision oncology.

The finding that patients aged 50 or older had 79% lower odds of receiving testing (**Table 4**) aligns with current guidelines, such as ASCO, which historically prioritized testing recommendations for younger patients or for specific subgroups such as triple-negative breast cancer, whose immediate surgical management is more likely to change with a positive result (25).

Importantly, the 71.7% rate of change in treatment plans, and willingness to test other family members (77.1%) indicates the clinical utility of the test, leading to more personalized and potentially more effective management strategies. Therefore, we advocate for the incorporation of *BRCA1/2*, testing into the mandatory procedures within the SUS in order to waive the socioeconomic disparities regarding access to genetic testing in Brazil, allowing it to become the standard of care for all eligible patients. Expanding the private testing criteria to the public system, although still not ideal, should be an important initial step to reduce the disparities between the two systems.

Expanding test coverage, however, would be insufficient without addressing the professional capacity to implement it, as the management of the test results is dependent on qualified professional guidance (26). Given the recognized structural shortage of medical geneticists in Brazil, limiting breast cancer genetic testing to geneticists may restrict access and delay care. Thus, we also advocate for the development and implementation of training programs aimed at oncologists, surgeons, and mastologists, equipping this non-genetic workforce with skills to act in line with oncogeneticists, ensuring that in regions or locations with a shortage of the professionals, other specialties that are part of multiprofessional team, can guarantee the request of the tests, as well as the correct interpretation of the results and pre and posttest counseling.

## 5 Study limitations

We acknowledge several limitations in this study that must be considered when interpreting the results. First, the most significant limitation is the inherent selection bias. The recruitment strategy, primarily leveraging patient advocacy groups and digital platforms, resulted in a cohort with a substantially higher socioeconomic and educational status than the average Brazilian population, with a heavy concentration in the Southeast region. Additionally, a formal *a priori* sample size calculation to determine statistical power was not performed, and we were unable to assess how many patients applied to the questionnaire in total, potentially leading to non-respondent bias where patients who did not complete the survey have different characteristics than patients who submitted the questionnaire, potentially selecting patients with a higher level of engagement among survey respondents, introducing self-selection bias in the answers. Therefore, we acknowledge that the lack of internal and external validity inherent to this type of open, voluntary survey design prevents the generalization of these findings to the broader Brazilian context. Instead, these results must be interpreted as a case study representing the experiences of a highly-engaged patient group, with access to healthcare resources.

Second, the survey instrument itself presents methodological limitations. Although part of a global study, the questionnaire was not psychometrically validated specifically for the Brazilian cultural and healthcare context. A key deficiency in the instrument was its failure to include questions differentiating patient experiences between the public system (SUS) and the private supplementary health system. Because this questionnaire was standardized to be applied in an international setting, it did not collect data to differentiate whether participants received care predominantly through SUS or the private healthcare system in Brazil.

Third, oncologists played a central role in the genetic testing process for Brazilian patients (27). However, due to the requirement for international standardization of the questionnaire, the survey did not include mastologists in response options of questions 11 and 12. In Brazil, mastology is a specialty trained to prevent, diagnose, and treat conditions of the breasts (28). Since the most common change in treatment was the switch to bilateral mastectomy, which is a therapeutic intervention typical of surgeons, we hypothesize that results could have been different if mastologists were included in questions 11 and 12 of the survey.

Finally, the survey’s cross-sectional and descriptive design limits our ability to establish any causality. While we identified significant associations, we cannot determine a direct and temporal relationship. Furthermore, these associations should not be generalized to broader populations beyond the sample of the survey.

## 6 Final remarks

In summary, the analysis of the Brazilian MAGENTA study cohort, composed mainly of patients with high educational and socioeconomic status, demonstrates that, when informed and with access to resources, the breast cancer patient becomes an active agent in her treatment journey and in their family’s preventive care. The results of this survey offer a glimpse into the transformative potential of breast cancer genetic testing. Even in this highly selected cohort of patients, we found that age and income level were independently associated with a higher odds of taking breast cancer genetic testing. Since testing is only warranted in the private health system, our findings suggest that high costs and insurance coverage are the main barriers to testing, and therefore should be the targets of public policies aiming to reduce the disparities in the access to breast cancer genetic testing in Brazil. Therefore, this study serves as a call to action for public policies and health sector initiatives to work towards extending the benefits demonstrated here to all Brazilian women.

## Data Availability

The original contributions presented in the study are included in the article/**Supplementary Material**. Further inquiries can be directed to the corresponding author.
